# The contribution of semantics to the sentence superiority effect

**DOI:** 10.1038/s41598-021-99565-6

**Published:** 2021-10-11

**Authors:** Stéphanie Massol, Jonathan Mirault, Jonathan Grainger

**Affiliations:** 1 Laboratoire d’Etude des Mécanismes Cognitifs, University of Lyon2, Lyon, France; 2 Laboratoire de Psychologie Cognitive, Centre National de Recherche Scientifique & Aix-Marseille University, Marseille, France; 3 Laboratoire d’Étude des Mécanismes Cognitifs (EMC), Institut de Psychologie, Université Lumière Lyon 2, 5 Avenue Pierre Mendès France, 69676 Bron Cedex, France

**Keywords:** Human behaviour, Psychology

## Abstract

When a sequence of written words is presented briefly and participants are asked to report the identity of one of the words, identification accuracy is higher when the words form a correct sentence. Here we examined the extent to which this sentence superiority effect can be modulated by semantic content. The central hypothesis guiding this study is that the sentence superiority effect is primarily a syntactic effect. We therefore predicted little or no modulation of the effect by semantics. The influence of semantic content was measured by comparing the sentence superiority effect obtained with semantically regular sentences (e.g., *son amie danse bien* [her friend dances well]) and semantically anomalous but syntactically correct sentences (e.g., *votre sac boit gros* [your bag drinks big]), with effects being measured against ungrammatical scrambled versions of the same words in both cases. We found sentence superiority effects with both types of sentences, and a significant interaction, such that the effects were greater with semantically regular sentences compared with semantically anomalous sentences. We conclude that sentence-level semantic information can constrain word identities under parallel word processing, albeit with less impact than that exerted by syntax.

## Introduction

The sentence superiority effect, first reported by Cattell^1^ (reported in Scheerer^2^), and more recently investigated in memory research^3^, has attracted renewed interest following the finding that the effect can be observed under conditions of Rapid Parallel Visual Presentation (RPVP) of word sequences^4^. The standard finding is that free report of a sequence of words is better when the sequence forms a correct sentence compared with ungrammatical sequences. Drawing inspiration from research on the word superiority effect [^5,6^, see^7^ for a review], Snell and Grainger^4^ used the RPVP paradigm combined with post-cued report of a single word in the sequence in order to rule-out memory contributions to the sentence superiority effect. Snell and Grainger found that single word identification accuracy was significantly greater when the word was presented in a grammatically correct context (e.g., the target word “man” in “the man can run”) compared with an ungrammatical sequence of the same words (e.g., “ran man the can”).

Following this report of a sentence superiority effect using the RPVP procedure it has been shown that: (1) the effect is also obtained in primary school children in Grade 3 (average age 8.9 years)^8^, and (2) that the effect emerges around 300 ms post-sequence onset in EEG recordings^9^. The latter finding points to a key role for interactions between sentence-level structures and on-going word identification processes in driving the sentence superiority effect. More generally speaking, these findings provide support for a cascaded, interactive model of sentence comprehension according to which the rapid partial processing of multiple word identities provides sufficient information to activate sentence-level structures, which in turn provide feedback to on-going word identification processes^9^. The key question then is the nature of the sentence-level representations that are involved in this initial bottom-up activation process. That is, to what extent do syntactic and semantic representations contribute to the construction of a primitive sentence-level representation that is crucial for rapid, efficient sentence comprehension (cf. the notion of “good-enough” sentence representations:^10^).

One important finding with respect to this central question was reported by Declerck et al.^10^. In that study, French–English bilinguals were presented with word sequences composed of a mixture of French and English words. For example, a “grammatically correct” sequence would be formed of French and English words that, when translated, would generate a correct sentence in either language (e.g., “ses feet sont big”, which after translation gives “ses pieds sont grands”/“his feet are big”). Accuracy in identifying a given target word (e.g., feet) was then compared with identification of the same word at the same position in a scrambled version of the same set of words (e.g., the target word “feet” in “sont feet ses big”). Declerck et al.^10^ found a robust sentence superiority effect in these conditions and concluded that the effects were driven by the parallel processing of word identities and the association of these word identities with language-independent syntactic categories (e.g., noun, verb). This was hypothesized to enable the rapid construction of a primitive language-independent syntactic structure that would then provide feedback to on-going word identification processes.

These findings provided important support for a model of reading first proposed by Snell et al.^11^ and developed essentially on the basis of findings obtained with a different paradigm—the reading version of the flankers task. In this paradigm, a central target word is presented simultaneously, and briefly (170 ms), accompanied by stimuli to the left and right (the flankers) and separated by a single space as in normal text. Participants are requested to respond to the central targets and ignore the flankers. Orthographic^12,13^ and syntactic^11^ flanker effects were taken to infer that: (1) orthographic information (letter identities and letter order) spanning several words can be processed in parallel and this information is pooled into a single channel for word identification; (2) several word identities can be processed in parallel; and (3) syntactic category information can be retrieved in parallel from multiple words [see^14,15^ for reviews]. Snell et al.^11^ completed this parallel processing perspective with feedback from a sentence-level syntactic representation to on-going word identification processes. Thus, in the Snell et al.^11^ model, the parallel identification of word identities and the associated retrieval of syntactic category information enables the rapid computation of an approximate syntactic structure (i.e., good-enough syntax^16^; or a first-pass syntactic parse^17^), and this syntactic structure is used to constrain on-going word identification processes in a parallel, cascaded, interactive manner^9^.

According to the Snell et al.^11^ model, the sentence superiority effect should be primarily driven by syntax, and the contextually driven expectation that a word at a given position should belong to a certain syntactic category (i.e., a syntactic rather than a semantic prediction). However, when interpreting their observation of a sentence superiority effect, Snell and Grainger^4^ left open the possibility that sentence-level representations, other than a purely syntactic representation, might play a role. In other words, sentence-level representations could impact on word identification via feedback influences, be they syntactic or semantic in nature (see also Snell et al.^18^, for evidence in favor of this potential extension of the Snell et al.^11^, model).

As a test of a purely syntactic interpretation of the sentence superiority effect versus a more general sentence-level effect, we therefore designed an experiment in order to examine a potential role of semantics in modulating this effect. In order to do so, we compared the size of the sentence superiority effect obtained with semantically regular sentences such as “*son amie danse bien*” [her friend dances well] compared with semantically anomalous sentences such as “*votre sac boit gros*” [your bag drinks big]. In both cases, the size of the sentence superiority effect was measured by comparing identification of the same target word at the same position in an ungrammatical sequence of the same set of words (e.g., target “amie” in “danse amie bien son”, and the target “sac” in “boit sac votre gros”). The goal of the present study was therefore to compare the size of the sentence superiority effect obtained with semantically regular sentences and semantically anomalous but syntactically correct sentences in order to estimate the relative contribution of syntax and semantics to sentence superiority effects. Participants saw sequences of four words that either formed a correct sentence or a scrambled ungrammatical sequence of the same words and were asked to report the identity of the word at a post-cued location that varied randomly from trial-to-trial.

## Results

### Main analysis

**Figure 1:**
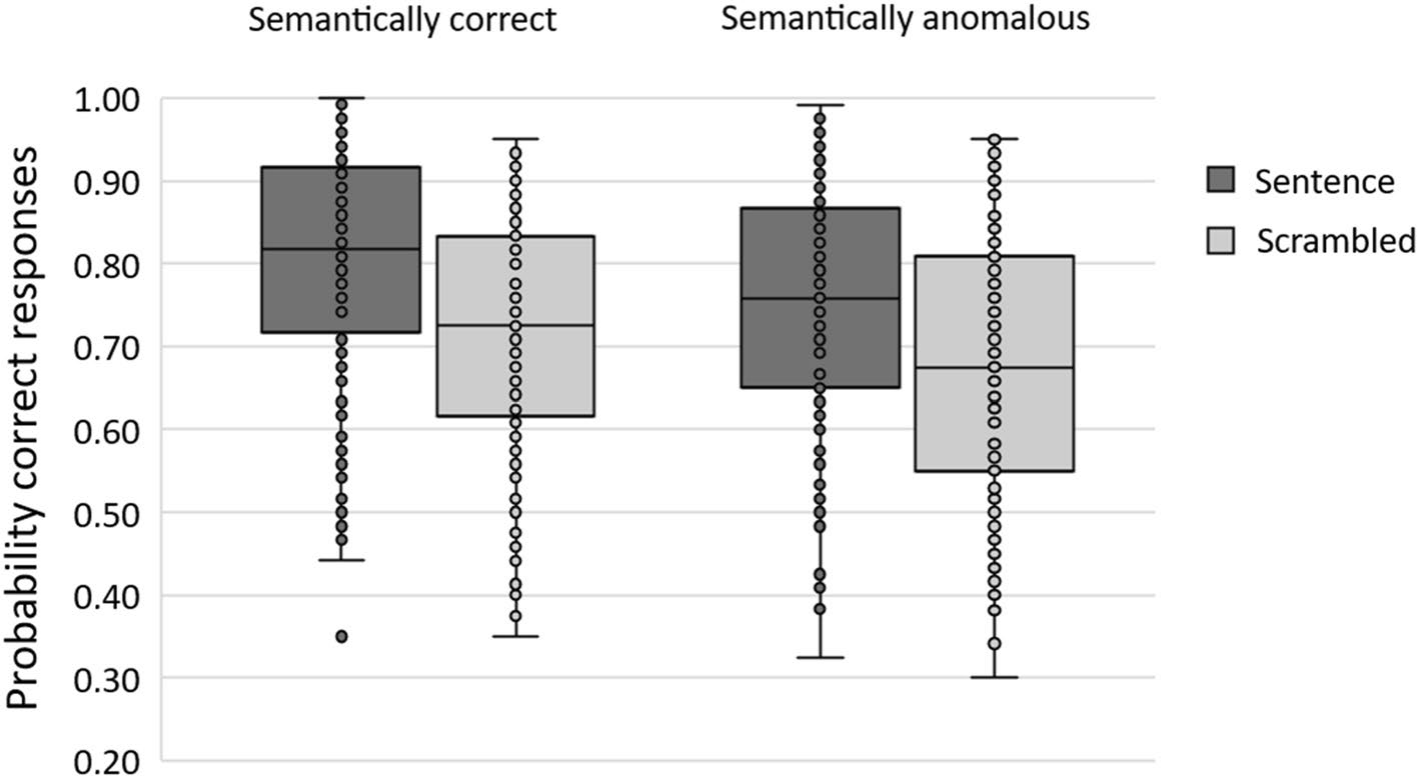
Box-and-whisker plot of the sentence superiority effect (sentence vs. scrambled) obtained with semantically regular and semantically anomalous sentences. Within each box, the horizontal lines denote the median value and the box boundaries indicate the 25th and 75th percentiles. The whiskers mark the 5th and 95th percentiles and each data point represents individual participant means.

We used Generalized (logistic) Linear Mixed-Effects models (LMEs) to analyze our data, with items and participants as crossed random effects and including by-item and by-participant random intercepts and random slopes^19^. The logistic mixed-effects model included Syntax (correct sentences vs. ungrammatical scrambled sequences of the same words) and Semantics (semantically regular sentences vs. semantically anomalous sentences) as fixed-factors, and Position (1–4) and Word Length (in number of letters) as covariables. Note that the main effects of Position and of Word Length were not of interest here (see Supplementary Fig. S1 for the results broken down by position). The models were fitted with the glmer function from the lme4 package^19^ in the R statistical computing environment (version 4.0.2^20^). The maximal random effects structure that converged was one including by-participant and by-item random intercepts. The following analyses were conducted taking the correct sentence condition as reference for the Syntax factor and the semantically regular sentence condition as reference for the Semantics factor. We report regression coefficients (*b*), standard errors (SE) and z-values for all factors. Fixed effects were deemed reliable if |z| > 1.96^21^. The condition means of probability of correct responses are shown in Fig. 1.

Word identification accuracy was greater in the correct sentence condition than in the scrambled word sequence condition (*b* = − 0.63, *SD* = 0.02, *z* = − 23.07). Moreover, there was a significant effect of Semantics, with targets presented in the semantically regular sentences being identified more accurately than targets presented in semantically anomalous sentences (*b* = − 0.41, *SD* = 0.09, *z* = − 4.56). The interaction between Syntax and Semantics was significant (*b* = 0.16, *SD* = 0.03, *z* = 4.45). Follow-up analyses revealed that the effect of Syntax was highly significant in both the semantically regular sentences (*b* = − 0.63, *SD* = 0.02, *z* = − 22.99) and the semantically anomalous sentences (*b* = − 0.46, *SD* = 0.02, *z* = − 17.92), but with the effect of Syntax being greater in the semantically regular sentence condition (see Fig. 1). Note that the effect of Position was significant (*b* = − 0.33, *SD* = 0.03, *z* = − 8.36) as well as the effect of Word Length (*b* = 0.14, *SD* = 0.05, *z* = 2.50).

### Post-hoc analyses

These additional analyses were designed to examine any potential impact of target word frequency, type of syntactic structure, and participants’ age (given the evidence that age impacts on syntactic and semantic processing;^22^) on the pattern of results obtained in the main analysis. Target word frequency was treated as a binary variable (high vs. medium), syntactic structure was encoded using dummy coding (i.e., arbitrarily assigning a number to the different syntactic structures: 1–15), and age was entered as a continuous covariable. Note that the glmer analysis failed to converge when target word frequency was treated as a continuous covariable. The details of these analyses are provided in the “Supplementary Materials S1”. The key findings are that in all three post-hoc analyses the pattern seen in the main analysis replicated, with main effects of Syntax and Semantics, and a significant interaction. The only additional finding was that target word frequency interacted significantly with Syntax (*b* = 0.40, *SD* = 0.05, *z* = 7.09), reflecting the greater sentence superiority effect with high-frequency target words (see Figure S2 in the “Supplementary Materials S1”). Although we found the same pattern of effects after controlling for syntactic structure, we note here that an interesting avenue for future research would be to investigate the impact of syntactic complexity (e.g., active vs. passive structures) on the sentence superiority effect.

## Discussion

The present study was designed to measure the contribution of semantics to the sentence superiority effect. This was motivated by both theoretical^11^ and empirical^10^ considerations pointing to a uniquely syntactic interpretation of sentence superiority. The sentence superiority effect is the observed superior identification of a post-cued word in a briefly presented sequence of words when the sequence is syntactically correct (e.g., *son amie danse bien* [her friend dances well]) compared with ungrammatical scrambled sequences of the same words (*danse amie bien* son [dances friend well her]). This finding provides support for a cascaded, interactive account sentence comprehension according to which partial word identification processes operating in parallel enable the rapid activation of a sentence-level representation that then provides feedback to on-going word identification processes^9,11^. The present study investigated the relative contribution of syntactic and semantic information in the activation of an initial “good-enough” sentence-level representation^23^.

The results of Declerk et al.^10^ suggested that the sentence superiority effect is essentially driven by word identities activating their corresponding parts-of-speech (e.g., “son” [her] = determiner) which enables the rapid computation of a primitive syntactic structure. It is this syntactic structure that then provides constraints on possible word identities at a given position, when the word sequence is syntactically correct. In other words, the initial “good-enough”^23^ sentence-level representation would be essentially a syntactic representation. However, syntactically correct word sequences are also more semantically coherent than ungrammatical word sequences. Indeed, Snell and Grainger^4^ had already noted the possible role played by sentence-level semantic information in driving the sentence superiority effect (see Snell et al.^18^, for evidence for the parallel activation of semantic information across multiple words). Thus, part of the effect attributed to syntax in our prior work, could be driven by sentence-level semantic representations. Therefore, in order to evaluate the contribution of semantics to the sentence superiority effect we compared the size of the effect obtained with semantically regular sentences and semantically anomalous but syntactically correct word sequences. We found a significant contribution of semantics, with the size of the sentence superiority being significantly larger with semantically regular sentences compared with the semantically anomalous sentences. This pattern of findings fits with the tentative conclusions offered by Snell and Grainger^4^ when comparing their findings with those obtained in Japanese^24^ (albeit with a slightly different paradigm). In other words, sentence-level structures, be they syntactic or semantic, would constrain on-going word identification processes, and the relative impact of syntax and semantics is likely to be language-specific. One common factor here could well be predictability, and in line with this proposal is our finding that the sentence superiority effect was greater for high-frequency words, since word frequency provides a context-free measure of predictability.

Finally, it is important to note that the contribution of semantics to the sentence superiority effect, observed in the present study, was relatively small. In the semantically regular sentences, the size of the sentence superiority effect was 11.80%. In the semantically anomalous sentences, the size of the sentence superiority effect was 9.86%. Therefore, relative to the grand average sentence superiority effect (10.83%) semantics contributed 19.7%. This is a relatively small contribution that could be due to the fact that our semantically regular sentences were selected to have relatively low constraint (i.e., low cloze probabilities). Therefore, future studies could investigate the contribution of semantics to sentence superiority by manipulating the cloze probability of words at different positions in the syntactically correct sentences (i.e., a parallel version of the cloze test). This cloze probability manipulation would provide a measure of semantic predictability (e.g., this word is likely to be an animated noun) as opposed to a purely syntactic predictability (e.g., this word must be a noun).

## Methods

### Participants

A total of 180 participants (94 male) with a mean age of 28.5 years (SD = 9.46, min–max: 18–72-years-old) were recruited through on-line advertisements. All participants were native speakers of French, with no history of neurological impairment, or developmental disorders. The number of participants we recruited was based on the criterion proposed by Brysbaert and Stevens^25^ and provided a statistical power of 99.80% (95% CI 0.35). Note that we used the SMIR package from Green and MacLeod^26^ with 1000 simulations on the semantically correct sentences to find the simple effect of syntax. Prior to the beginning of the experiment, participants were informed that data would be collected anonymously, and they provided informed consent before the experiment was initiated.

### Materials

240 grammatically and semantically regular sentences were constructed. They all consisted of four French words each. Word length was from three to five letters, and the summed frequency of the content words was 5.66 Zipf (SD = 0.7; log10 (occurrences per million words) + 3^27^). We also constructed another set of 240 grammatically correct sentences that were semantically anomalous (e.g., *votre sac boit trop* [your bag drinks big]). The semantic anomaly could be either between the subject and the verb (e.g., *votre avion rêve peu* [your plane dreams little], 80 sentences), between the subject and the complement (e.g., *ton matin est gros* [your morning is fat], 80 sentences), or between the verb and the complement (e.g., *pépé mange ses sous* [grandpa eats his coins], 80 sentences). Similar to the first set of sentences, these sentences consisted of four words each, with word length between three to five letters, and the summed frequency of the content words was 5.58 Zipf (SD = 0.73). To ensure that the 240 sentences were low constraint (i.e., words were not easily predictable given the sentence context), a cloze test was performed with an independent group of 113 participants in order to measure cloze probability of the last word for both the semantically regular and semantically anomalous sentences. The participants were divided in two groups (group 1 = 55 participants, group 2 = 58 participants), and each group saw a total of 240 sentences (120 regular sentences and 120 anomalous sentences). These measures were collected through an on-line experiment (Google Forms) which consisted of a sentence completion test. Participants were presented with the beginnings of the sentence (i.e., the first three words) and were asked to type the first word that came to their mind as a likely continuation of the sentence. Cloze probability was calculated as the number of answers that corresponded to the word at position 4 in the original sentence divided by the number of participants. The average cloze probability of these words was 0.05 (SD = 0.11) for regular sentences and 0.01 (SD = 0.04) for anomalous sentences. We also checked the degree of semantic plausibility of these sentences, again on-line using Google Forms. Participants were divided in two groups (group 1 = 59 participants, group 2 = 53 participants), and each group judged a total of 240 sequences (120 regular sentences and 120 anomalous sentences, randomly intermixed). Sentences were presented to the participants and they were asked to evaluate whether the sentence was semantically plausible or not, using a scale from 1 (semantically nonsense) to 5 (semantically regular). The average rating for the regular sentences was 4.70 (SD = 0.32) and was 1.63 (SD = 0.04) for the anomalous sentences.

For the purpose of the word-in-sequence identification task, one of the four words in every sentence was marked as the critical target at one of the four possible positions, such that there were 30 critical targets for each position. Finally, we constructed grammatically incorrect word sequences based on these two sets of 240 sentences by scrambling word order in the correct sentences but keeping the target word in the same position (e.g., the target word “amie” in *son amie danse bien* [her friend dances well] versus *danse amie bien son* [dances friend well her]). These two sets of 240-word sequences were separated into two subsets to create two lists of experimental stimuli presented to different participants. Grammatically correct sentences presented in one list were paired with their scrambled ungrammatical version in the other list, and vice versa. In this way, participants saw each sentence only once but were tested in all conditions with different sentences. Across participants, each set of words in a 4-word sequence occurred only once either in its grammatically correct version (e.g., *son amie danse bien*) or in the ungrammatical scrambled version (e.g., *danse amie bien son*).

### Procedure

**Figure 2:**
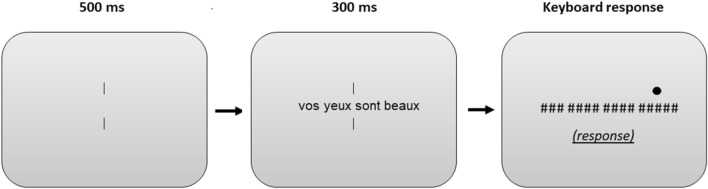
The post-cued partial report rapid parallel visual presentation procedure used in the present experiment. Participants had to type in the word they thought had been presented at the post-cued location, indicated by a filled circle.

Participants performed this task on-line. Stimuli were presented using HTML, PHP and Java protocols on the personal computer screen of the participant. All sequences were presented as black letters in lowercase (font size = 28) and centered vertically and horizontally on a light grey background. Written instructions were given at the beginning of the experiment. Participants were informed that a sequence of four words was going to be displayed briefly and followed by a pattern mask. They were asked to decide which word they had seen in the position indicated by a dot above the target location that accompanied the pattern mask (i.e., a post-cue). Each trial began with the presentation of two vertical bars positioned at the center of the screen and that remained on the screen during the whole trial duration. Participants were asked to focus their attention on the center between the two vertical bars at the beginning of each trial. 500 ms later, the sentence was centrally displayed during 300 ms (i.e., two words appeared to the left and two to the right of fixation, with equal and normal between-word spacing). This was then followed by a backward mask composed of hash marks at all positions that were occupied by a letter in the previous string and accompanied by the post-cue for the target word location (i.e., the dot above the target location). Participants could type their response at this point, and their response appeared in a box located slightly below the string of hash marks. Once the return key was pressed, the next trial was displayed (Fig. 2). A practice session (16 trials) was administered before the main experiment to familiarize participants with the procedure. The 480 trials of the main experiment were presented in a random order. The experiment lasted approximately 25 min.

### Data analysis

We first screened the data and excluded 17 participants prior to further analysis. Two of these participants were excluded due to misunderstanding the instructions (their responses contained several words rather than a single word), three participants did not finish the experiment, and 12 participants were excluded based on their low level of overall accuracy (i.e., accuracy below 2.5SD from the grand mean—less than 35.27%). This left a total of 163 participants (85 males, mean age = 28.63 years, SD = 9.49) that were included in the final analysis. Second, in order to provide an objective analysis of participants’ responses (i.e., error categorization), all responses were first checked by an algorithm developed in order to automatize this process^28^. Finally, a human observer (one of the authors) checked the output of the algorithm. The final dataset was composed of 78,218 observations. The glmer model used in the main analysis was the following: Correct ~ Semantics*Syntax + Position + Length + (1|Subject) + (1|Item).

### Ethical statement

The study was approved by ethics committee of Comité de Protection des Personnes SUD-EST IV (No. 17/051).

## Supplementary Information

Supplementary Information.

## Data Availability

The authors confirm the availability of shared data. The dataset and analysis scripts used with R are accessible on the OSF website at: https://osf.io/7wf5k/.
