# Supplementary material for: The contribution of semantics to the sentence superiority effect
Source: Sci Rep. 2021 Oct 11;11:20148. doi: 10.1038/s41598-021-99565-6 (PMC8505490; doi:10.1038/s41598-021-99565-6)
Supplement: Supplementary file 1 — Supplementary Information. [file 41598_2021_99565_MOESM1_ESM.docx]

**Supplementary Materials**

Here we report the details of results as well as additional analyses that were only briefly mentioned in the manuscript.

*Target word position*

**
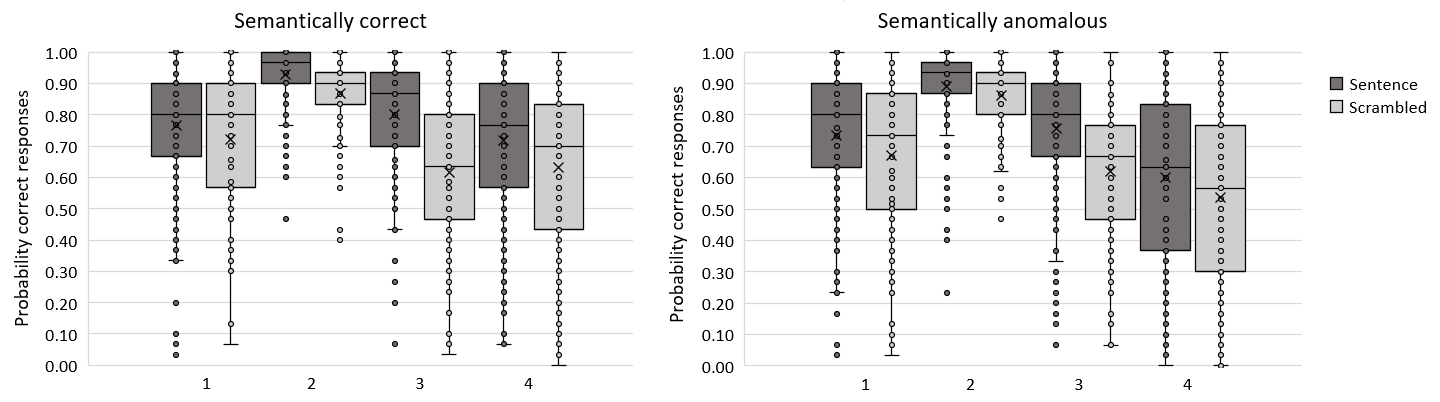
**

*Figure S1*. Box-and-whisker plots of the sentence superiority effect at the different target positions (1-4) in the syntactically correct sentence conditions (Sentence) and in the ungrammatical scrambled word sequence conditions (Scrambled) for the semantically regular sentences (left panel) and for the semantically anomalous sentences (right panel). Within each box, the horizontal line denotes the median value and the black cross the mean value, and the box boundaries represent the 25th and 75th percentiles. The whiskers mark the 5th and 95th percentiles, and each data point represents individual participant means.

*Target word frequency*

In order to test for an influence of word target frequency on the main effects of Syntax and Semantics and their interaction, we divided the sentences into two sets according to the frequency of the target word. In this data set, a total of 452 sentences were selected based on a median split (median target word frequency = 5.41 Zipf). 226 sentences were classified as “medium-frequency” (mean = 4.48 Zipf, SD = 0.76), and 226 sentences as “high-frequency” (mean = 6.10 Zipf, SD = 0.49). We then ran an analysis that included Frequency as a two-level factor: Correct ~ Semantics * Syntax * Frequency + (1|Subjet) + (1|Item).

There was a main effect of Syntax (*b* = -0.87, *SD* = 0.04, *z* = -21.00), a main effect of Semantics (*b* = -0.40, *SD* = 0.14, *z* = -2.87) and a significant Syntax X Semantics interaction (*b* = 0.40, *SD* = 0.05, *z* = 7.09). Moreover, the interaction between Syntax and Frequency was significant (*b* = 0.40, *SD* = 0.05, *z* = 7.09). As can be seen in Figure S2, the effect of Syntax was greater for high-frequency words (*b* = -0.84, *SD* = 0.04, *z* = -20.55) as compared to medium-frequency words (*b* = -0.47, *SD* = 0.04, *z* = -11.74). Crucially, the three-way interaction was not significant, *b* = 0.12, *SD* = 0.07, *z* = 1.51.


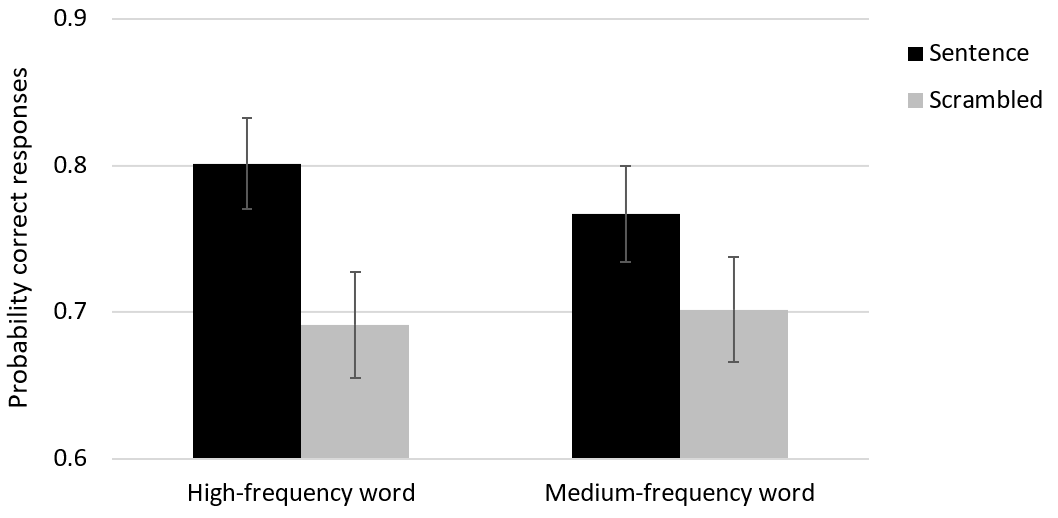


*Figure S2*. The sentence superiority effect (sentence vs. scrambled) obtained in the high-frequency target word condition (left panel) and in the medium-frequency target word condition (right panel). Error bars are standard errors.

*Syntactic structure*

From the complete stimulus set, we selected 419 sentences that had a syntactic structure (i.e., a specific sequence of syntactic categories: determiner, noun, verb, etc.) that occurred in both the semantically correct sentences (196 sentences) and the semantically anomalous sentences (223 sentences). We then ran an analysis with Syntactic Structure entered using dummy coding for the 15 different structures. The model was the following: Correct ~ Semantics * Syntax + SyntacticStruc + (1|Subjet) + (1|Item). As can be seen in Figure S3, we observed the same pattern of results as in the main analysis, with a main effect of Syntax (*b* = -0.59, *SD* = 0.03, *z* = -19.93), a main effect of Semantics (*b* = -0.43, *SD* = 0.10, *z* = -4.08) and a significant Syntax X Semantics interaction (*b* = 0.14, *SD* = 0.04, *z* = 3.46).


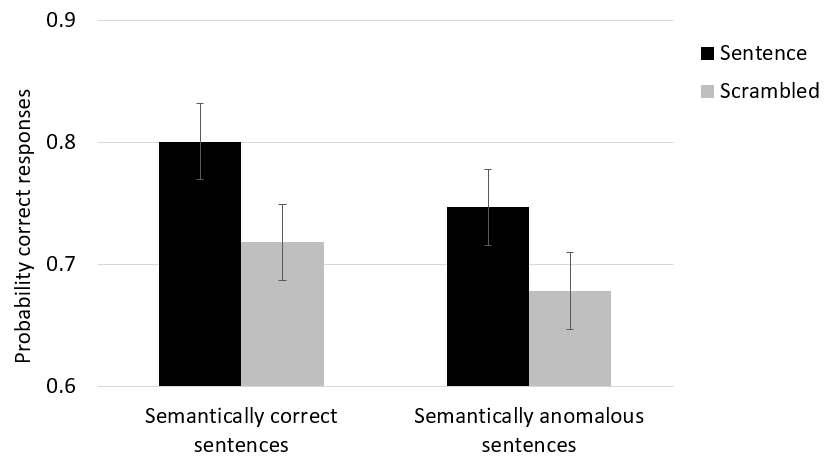


*Figure S3*. The sentence superiority effect (sentence vs. scrambled) obtained in the semantically correct sentences (196 sentences) and in the semantically anomalous sentences (223 sentences) when matched for syntactic structure. Error bars are standard errors.

*Age of participants*

We ran a further analysis taking into account Syntax and Semantic as fixed-factors and Age as covariable. The results revealed the same pattern of results as in the main analysis, with a main effect of Syntax (*b* = -0.63, *SD* = 0.02, *z* = -20.06), a main effect of Semantics (*b* = -0.41, *SD* = 0.09, *z* = -4.26) and a significant interaction between Syntax and Semantics (*b* = 0.16, *SD* = 0.03, *z* = 4.45). The main effect of Age was marginally significant, *b* = -0.01, *SD* < 0.01, *z* = -1.73.
